# Para and Post-COVID-19 CNS Acute Demyelinating Disorders in Children: A Case Series on Expanding the Spectrum of Clinical and Radiological Characteristics

**DOI:** 10.7759/cureus.23405

**Published:** 2022-03-22

**Authors:** Abdulhafeez M Khair, Rahul Nikam, Sumair Husain, Melanie Ortiz, Gurcharanjeet Kaur

**Affiliations:** 1 Pediatric Neurology, Nemours Children's Hospital, Wilmington, USA; 2 Neuroradiology, Nemours Children's Hospital, Wilmington, USA

**Keywords:** a review, case report series, immune mediated phenomenon, post covid-19 manifestations, covid-19 in children, demyelinating neurological disorder

## Abstract

Viral infections can serve as a trigger for variable autoimmune, antibody-mediated demyelinating disorders. There is accumulating evidence that the severe acute respiratory syndrome coronavirus 2 (SARS-CoV-2) virus, causing coronavirus disease 2019 (COVID-19) infection and responsible for the current worldwide pandemic, can lead to a cascade of immune-mediated brain and spinal cord demyelinating injuries. However, such observation in the pediatric age group was only reported in very few patients. Thus, the heterogeneous spectrum of this phenomenon in children is still unfolding. We are reporting a case series of five pediatric patients with a variety of acute central nervous system (CNS) demyelinating disorders in the context of acute or recent COVID-19 infection. A 16-year-old female with anti-myelin oligodendrocyte glycoprotein (MOG) disorder, an eight-year-old male with acute disseminated encephalomyelitis (ADEM), a 13-year-old female with neuromyelitis optica spectrum disorder (NMOSD), and two 14 and 13-year-old females with new-onset multiple sclerosis (MS) are reported, all of whom presented acutely following COVID-19 infection. We propose that para and post-infectious CNS demyelinating disorders can potentially follow acute COVID-19 infection in children. Considering SARS-CoV-2 testing as a part of diagnostic workup is possibly useful. Awareness of the presence of this phenomenon can help in the recognition and management of those patients.

## Introduction

Para and post-infectious central nervous system (CNS) demyelinating disorders are rare but well-recognized groups of neurological disorders, typically attributed to preceding minor viral infections. The pathological hallmark is the destruction of dominantly white matter myelin and relative preservation of axons. Pathogenic pathways include targeted immune cells infiltration, maladaptive immune response, and variable contribution of cascades of inflammatory cytokines, often triggered by recent viral infections. Post-viral neurological symptoms have been repeatedly reported, for example, post-influenza, coronavirus, and measles.

The neurological manifestations of the severe acute respiratory syndrome coronavirus 2 (SARS-CoV-2) viral infection responsible for the current worldwide coronavirus disease 2019 (COVID-19) pandemic were observed in about one-third of patients. Headaches, acute encephalopathy, vascular strokes, and symptomatic seizures are among the commonly reported neurological symptoms in adult cohorts. Post-viral short and long-term neurological sequelae, including immune-mediated demyelinating disorders of the central and peripheral nervous systems have been reported in a few studies in the adult population. Isolated reports of acute demyelinating encephalomyelitis (ADEM), transverse myelitis (TS), and Guillain Barre Syndrome (GBS), for instance, are almost exclusively confined to adults. However, reports of particular correlation with the onset of CNS demyelinating disorders are sparse in the pediatric age group. Nevertheless, it is plausible to hypothesize that acute COVID-19 infection can trigger a series of immune-mediated sequelae in children, which may include various CNS demyelination syndromes. To our knowledge, this is one the largest, pediatric-specific case series of post-COVID-19 demyelinating disorders, highlighting the need for larger prospective studies to improve our understanding of this interesting phenomenon.

## Case presentation

A summary of the five cases in this series is provided in Table 1.

**Table 1:** Summary of the characteristics of the five reported patients IVIG: intravenous immunoglobulin; MOG: myelin oligodendrocyte glycoprotein; WM: white matter; NMOSD: neuromyelitis optica spectrum disorder

Patient	Age	Gender	Clinical presentation	Neuroimaging	Treatment	Diagnosis & Follow up
Case 1	16 y	F	Legs numbness, walking difficulty, blurring of vision	Hyperintensities within the white matter of both cerebral hemispheres	Steroids IV + oral, IVIG, then monthly IVIG for 6 months	Anti-MOG antibody demyelinating disorder, remarkable recovery, mild residual gait dysfunction
Case 2	8 y	M	Diplopia, imbalance, gait ataxia	Hyperintense lesions in the left pontomesencephalic junction, middle cerebellar peduncles, & right cerebellar WM	Steroids IV + oral, IVIG	Anti-MOG antibody demyelinating disorder, complete clinical recovery
Case 3	13 y	F	Headache, nausea, vomiting, dizziness, numbness, tingling, walking difficulty. Improved, then flare-up of all symptoms after COVID infection	Numerous hyperintense lesions throughout the brain, brainstem, cervical & thoracic spine	Steroids IV + oral	Relapsing NMOSD attributed to anti-Aquaporin-4 antibodies, moderate improvement, residual diffuse weakness
Case 4	14 y	F	Right leg weakness, left eye pain	Hyperintense, enhancing, and non-enhancing focal lesions in subcortical, periventricular, and deep WM, basal ganglia, hippocampi, optic chiasm, cervical & thoracic spine	Steroids IV + oral	New-onset pediatric multiple sclerosis, no follow up is available
Case 5	13 y	F	Headache, abdominal pain, blurring of vision, right-sided weakness, & walking difficulty	Hyperintense, enhancing, and non-enhancing lesions in cerebral WM, cerebellum, cervical & thoracic spine	Plasma exchange, steroids IV + PO, Rituximab	New-onset pediatric multiple sclerosis. Had one relapse that was responsive to steroids, then placed on Rituximab therapy

Case 1

**Figure 1:**
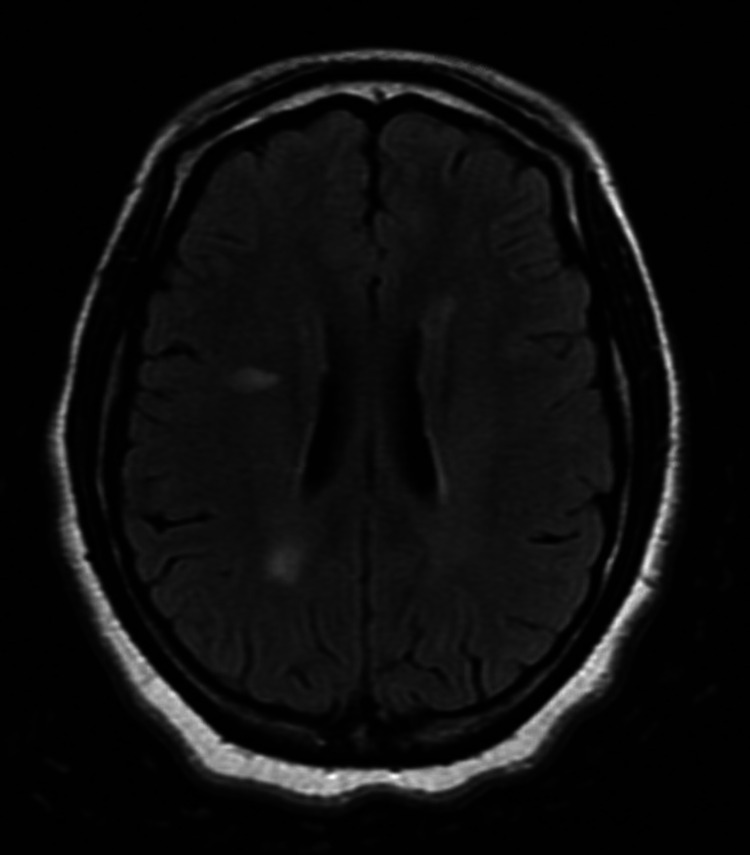
Case 1: axial T2/FLAIR sequence with foci of white matter hyperintensity suggestive of demyelination FLAIR: fluid-attenuated inversion recovery

A 16-year-old female with chronic lymphadenopathy presented with numbness and difficulty walking. Symptoms started in her feet one week prior to presentation. Over time, it progressed up to below her knees. She endorsed moderate pain in her feet during walking and was tripping frequently. She denied any change in bowel or bladder function. She also reported recurring episodes of having blurry vision and sometimes seeing complete black, usually lasting a few minutes with subsequent full recovery. She had contact with a known COVID-19-positive patient (her father), and she ultimately tested positive herself. Her bedside exam was mostly benign and only significant for brisk deep tendon reflexes (DTRs) at the patellae mainly, but with negative Babinski sign and no other long-tract motor findings. There was no dysmetria or ataxia, but on ambulation, her gait was mildly broad-based and seemed unsteady. Because of clinical concern for acute neurological decompensation, brain and spine MRI studies were urgently obtained. Brain MRI showed abnormal areas of increased T2 and fluid-attenuated inversion recovery (FLAIR) signal intensity within the white matter of both cerebral hemispheres (Figure 1). Spinal MRI showed mild contrast enhancement and increase signal at the T12 level. Extensive neuro-immunological workup revealed positive anti-MOG titers in serum and CSF studies. Testing for AQP4 antibodies was negative. CSF oligoclonal bands titers were mildly elevated. Interestingly, an expanded autoimmune encephalopathy panel also showed positive N-methyl-D-aspartate receptor (NMDA-R) antibodies in CSF but not in blood. She was treated and had a good clinical response to high-dose methylprednisolone followed by a tapering oral prednisolone course. Diagnosis of para-COVID-19 anti-MOG antibody demyelinating disorder was ultimately formulated. She was then placed on monthly IVIG therapy with good tolerance and relatively sustained improvement.

Case 2

**Figure 2:**
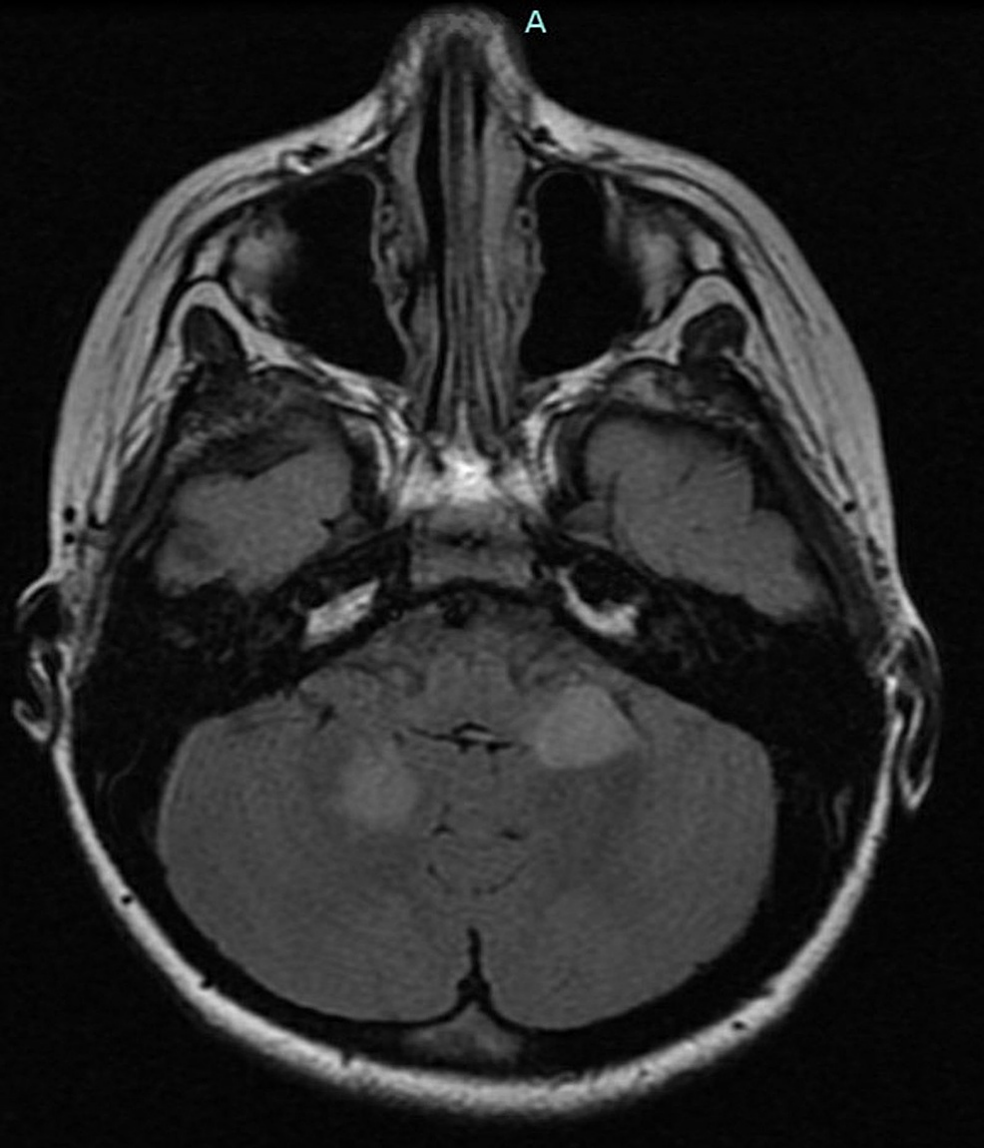
Case 2: T2/FLAIR hyperintense lesions in the middle cerebellar peduncles with extension to the cerebellar white matter FLAIR: fluid-attenuated inversion recovery

An eight-year-old male with no pertinent past medical history, presented with acute vision change and gait dysfunction. Two weeks prior to the presentation, he suddenly developed transient diplopia. The visual disturbance was intermittent for a couple of weeks. However, his mother started noticing gait abnormality that was consistent over the preceding two to three days. His family described that he seemed to be swaying and, at times, had to hold on to things to stay upright. No reported fainting, loss of consciousness, or syncopal episodes. He did not describe any significant weakness in his upper or lower extremities. His walking was described as if he had been "drinking". The family denied any possible exposure to alcohol or medications. His teachers at school noticed changes in his handwriting, and his mother confirmed that it did look different. In the emergency room, he also complained of a headache that he did not report at home. He denied having recent fever, cough, congestion, sore throat, vomiting, or diarrhea. His appetite was good. He did not sustain any head injuries or concussions in the past. Of note, the patient was diagnosed with acute COVID-19 infection about a month earlier although he only reported mild respiratory symptoms with no apparent complications or hospitalization needs. Upon evaluation, he was fully alert and appropriately interactive. However, he was noted to have consistent left-sided horizontal nystagmus on multiple evaluations. His gait was ataxic with normal patellar and ankle reflexes. No focal weakness or sensory deficits could be elicited. He had a head CT scan, which was unremarkable. Blood work for serum chemistry profile and urinary drug screening was normal as well. Contrast MRI studies were then obtained, which showed multiple, ovoid, T2/FLAIR hyperintense lesions within the left pontomesencephalic junction, left middle cerebellar peduncle with extension to the cerebellar white matter, right middle cerebellar peduncle, and right cerebellar white matter (Figure 2). Those lesions were accompanied by a peripheral rim of mild restricted diffusion and mild incomplete ring enhancement. Spinal cord and optic nerve MRI studies were normal. Testing for CNS infections, oligoclonal bands, immunoglobulin G (IGG) index, NMO antibodies, autoimmune encephalopathy panels, and MOG antibodies was negative for all. The patient received a course of 2 gram per kilogram IVIG and a 3-days-course of methylprednisolone with some clinical response. He was readmitted a few days later due to worsening gait dysfunction and ataxia and received another round of IVIG in addition to a five-day course of IV methylprednisolone followed by tapering oral prednisolone. Clinically, he improved with near resolution of his presenting symptoms and remarkable improvement of his ambulatory function. His working diagnosis at this point in time was post-COVID-19 ADEM variant or acquired demyelinating syndrome. 

Case 3

**Figure 3:**
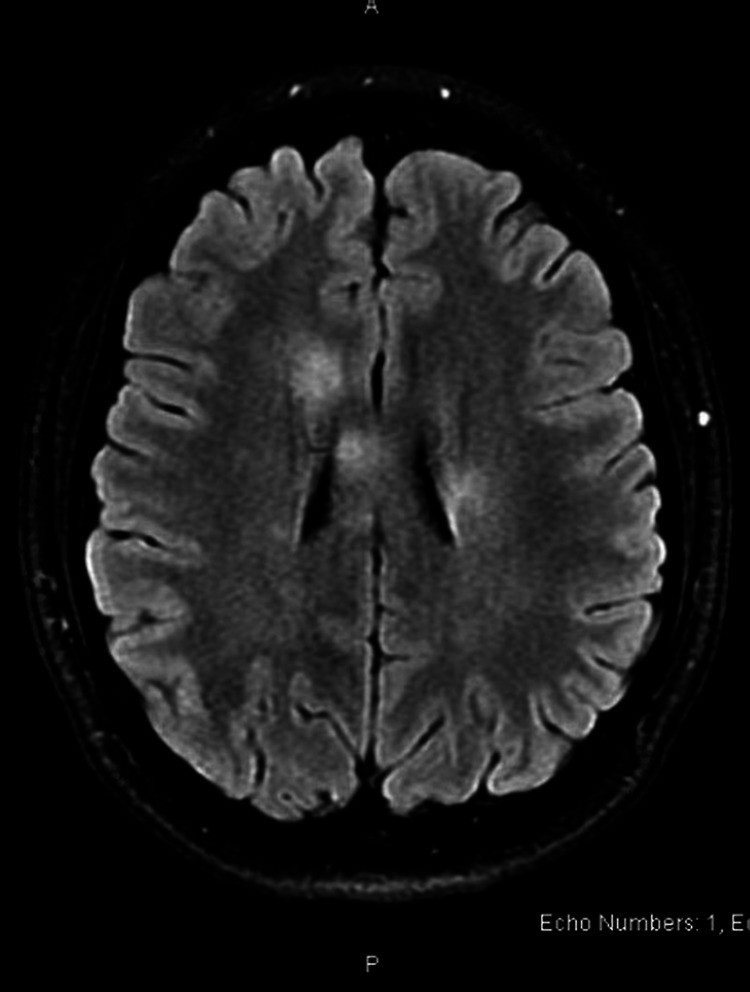
Case 3: Axial T2/FLAIR sequence showing asymmetric, non-enhancing, periventricular white matter hyperintensities FLAIR: fluid-attenuated inversion recovery

**Figure 4:**
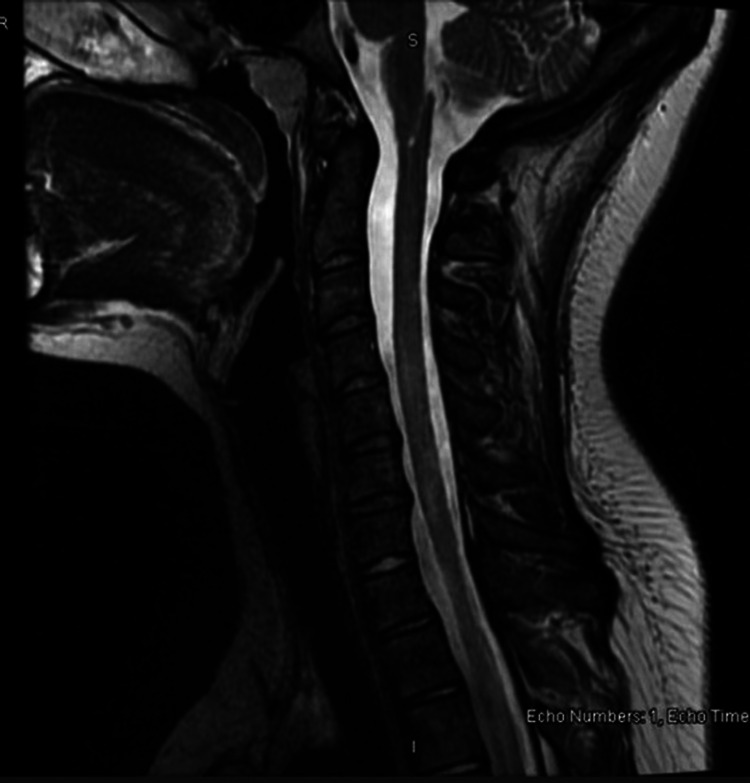
Case 3: Sagittal T2 sequence of the cervical and upper thoracic spine, showing abnormal hyperintense signals in the medulla and cervical spinal cord

A 13-year-old female with a past medical history of attention deficit hyperactivity disorder (ADHD) started experiencing new-onset headaches. These headaches were described as a "band" across her head and associated with light sensitivity, dizziness, and nausea with episodes of vomiting. Nausea and vomiting were particularly worse after waking up. She was significantly fatigued, taking frequent naps, having difficulty with ambulation, and needing to hold onto things for support. When walking, she described a left lower extremity pressure accompanied by numbness and tingling that radiated down her legs. She also endorsed having upper extremity weakness with difficulty brushing her teeth or hair. Additionally, she reported having double vision at times, without eye pain or redness. Otherwise, she denied fever, recent illnesses, dry eyes, dry mouth, joint swelling, joint pain, rashes, lymphadenopathy, constipation, or diarrhea. Brain MRI was ultimately obtained and showed asymmetric, non-enhancing, predominantly white matter lesions of the supratentorial and infratentorial brain with involvement of the medulla and upper cervical spinal cord, as well as poorly defined hyperintense foci in the lower spinal cord (T11-T12) extending through the conus. Workup for infectious etiology was negative. She was diagnosed with ADEM at the time and received a course of IV methylprednisolone followed by tapering prednisolone. Although all her symptoms improved, she did not retain all her baseline functions in the following months. Her follow-up MRI studies at six weeks were reported as normal. A month later, she was diagnosed with acute COVID-19 infection with the main symptoms of fatigue and loss of sense of smell and taste. About two months later, she had another MRI evaluation given the persistent fatigue and feeling of diffuse weakness and especially as she continued to test positive for the SARS-CoV-2 polymerase chain reaction test (PCR). The new MRI studies showed numerous patchy T2/FLAIR signal abnormalities throughout the brain, brainstem, and within the cervical and thoracic spinal cord (Figures 3-4). The distribution of these lesions in the brain and cervical spine was different from the very first MRI and the intracranial lesions appeared to be new, even in the absence of definite corresponding enhancement of any of the lesions. Further workup revealed very high titers of NMO antibodies in both serum and CSF samples. She also tested positive for the SS-B IgG antibody and the possibility of concomitant Sjogren was entertained. Testing for MOG, OCB, MBP, viral PCR panel, and autoimmune encephalopathy panel was negative. She was treated with another round of steroid therapy with symptomatic improvement. A diagnosis of para COVID-19 relapsing NMOSD attributed to anti-Aquaporin-4 antibodies was formulated.

Case 4

**Figure 5:**
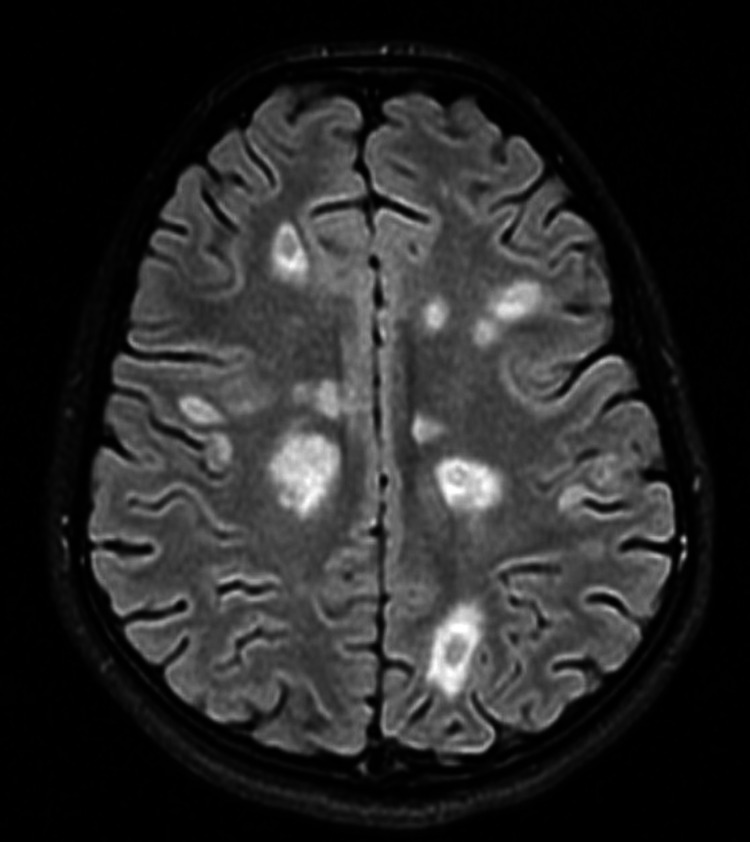
Case 4: Axial T2 MRI showing multiple, T2/FLAIR hyperintense, enhancing and non-enhancing focal lesions in the subcortical, periventricular, and deep white matter FLAIR: fluid-attenuated inversion recovery

A 14-year-old, previously healthy female presented with two weeks history of right leg pain in the groin area, which progressed to whole right leg weakness, as well as left eye pain with eye movement. She initially noticed pain in her right groin area and right calf when walking. No preceding trauma was reported. Over the course of a week, the pain progressed to include the entire right leg, and the patient developed subsequent weakness and numbness and became unable to ambulate independently. She tried over-the-counter naproxen for the pain at home, with some improvement. However, she also developed some intermittent left leg numbness as well. The patient also began having left eye pain, especially with an upward gaze. No eye redness or discharge was noted. There was no history of preceding eye trauma. No change in vision was associated with eye pain. Interestingly, she also developed acute urinary retention while being evaluated at the hospital. The patient had no bowel or bladder incontinence before hospitalization. She never had similar episodes of numbness, weakness, or eye pain in the past. No headaches, neck pain, change in behavior, confusion, or difficulty speaking were reported. Of note, the patient had exposure to a COVID-19 patient and a positive COVID-19 antigen test about five to six weeks prior to her presentation, although she was not symptomatic herself. Additionally, the family noted that she had a 13 lb weight loss over a period of two months. On examination, she was awake and alert and able to follow commands. She was noted to have a complete loss of strength in the right leg and partial weakness in the left leg but good strength in both arms. The sensory assessment demonstrated decreased sensation to fine touch and pinprick on the right lower extremity and coordination testing showed some subtle dysmetria of the right hand on the finger to nose test. A full brain and spine MRI was then obtained and showed multiple, T2/FLAIR hyperintense, enhancing and non-enhancing focal lesions in the supra and infratentorial brain parenchyma, within subcortical, periventricular, and deep white matter, bilateral inferior basal ganglia, and hippocampi (Figure 5). There was also an abnormal T2 hyperintense signal with an associated moderate postcontrast enhancement of left greater than right intra-orbital and pre-chiasmatic optic nerves, as well as multiple, T2 hyperintense, enhancing and non-enhancing intramedullary lesions in the cervical and thoracic spinal cord. Other studies showed increased CSF protein to 148 mg/dl, positive OCB, negative NMO antibodies, and negative CNS infection panels. MOG antibodies were not tested, and the patient had negative PCR for SARS-CoV-2 during hospitalization. She was treated with five days course of IV methylprednisolone followed by tapering with moderate clinical response. She was transferred to a rehabilitation unit thereafter. The patient was diagnosed with post-COVID-19 exposure new-onset multiple sclerosis. Unfortunately, follow-up on further clinical progress was lost.

Case 5

This was a 13-year-old female with no significant past medical history apart from COVID-19 exposure and PCR test positivity without reported symptoms at school a few weeks prior to presentation, needing only observation at home. She presented with a week history of headache, abdominal pain, vomiting, vision changes, right-sided arm and leg weakness, and difficulty with ambulation. She presented to the emergency department where she was given IV fluids, acetaminophen, and MiraLAX for presumed constipation. She reported some clinical improvement and was discharged home from the ED. However, a few days later, she developed right-sided weakness, along with occasional blurriness of vision upon looking to the left, and difficulty walking. These symptoms worsened, and on the day of admission, she went to her primary care physician. She had a fall at the primary doctor's office so she was sent back to the ED, where a stroke alert was initially called. An urgent brain MRI was done and showed Innumerable foci of increased T2 signal in the white matter of the brain, including the cerebellum and cervicomedullary junction. There was some enhancement of these lesions, some with ring enhancement and some with restricted diffusion suggesting lesions of different ages. Spinal MRI was then obtained and showed numerous small foci of T2 hyperintensity and enhancement involving the white matter of the spinal cord, mostly in the cervical spine on the right with one focus seen in the lower cord at the level of T11-12. The rest of the diagnostic workup showed positive CSF oligoclonal bands, high CSF IgG index, elevated CSF protein, negative viral PCR panel, negative NMO antibodies, and negative MOG antibodies titers. She was treated with IV methylprednisolone followed by oral prednisolone with good recovery. However, a repeated MRI surveillance three months later showed several FLAIR/T2 hyperintense lesions identified throughout the periventricular and subcortical white matter bilaterally. There were new FLAIR/T2 hyperintense lesions in the left temporal lobe, left occipital lobe, right frontal lobe, and right centrum semiovale, with many lesions again demonstrating contrast enhancement. She was readmitted, received a course of plasma exchange, IV methylprednisolone, and started on rituximab as a longer-term immunomodulator given the burden of her clinical and radiological lesions. Diagnosis of new-onset, severe pediatric multiple sclerosis following COVID-19 exposure was concluded.

## Discussion

**Figure 6:**
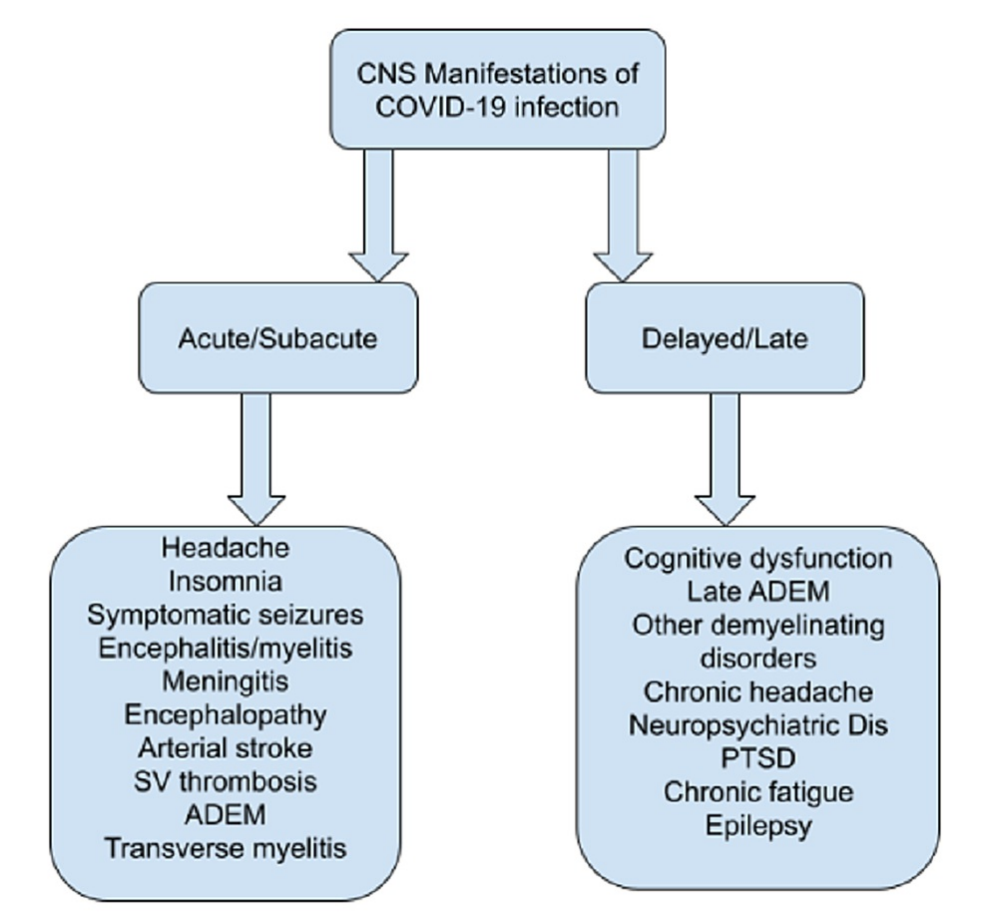
CNS neurological manifestations of COVID-19 ADEM: acute disseminated encephalomyelitis; PTSD: post-traumatic stress disorder; SV: superficial venous

The neurotropic and neuro-invasive potentials of the SARS-CoV-2 virus are being actively studied with multiple reports of distinct, mostly acute, neurological symptoms in patients with concurrent COVID-19 infections such as headaches, encephalopathy, strokes, neuropathy, and others [1]. Possible pathways of para infectious CNS dysfunction include direct brain invasion, hematogenous spread, neuronal migration, secondary hypoxic brain injury, angiotensin-converting enzyme dysfunction, autoimmune-mediated neurotoxicity, and inflammatory cascade pathways activation [2]. Exact pathophysiological mechanisms of viral-induced demyelinating injury are not well understood but are likely to involve either delayed cell-mediated or antibody-mediated cross-reaction to myelin basic protein, myelin oligodendrocyte protein, proteolipid protein, and other myelin autoantigens in CNS [3]. Mononuclear infiltration, inflammatory cytokine cascade production, and increased vascular permeability are other proposed mechanisms as well [4]. The resulting spectrum of clinical and neuroradiological manifestations is variable and includes post viral short and long-term neurological sequelae (Figure 6).

Acute disseminated encephalomyelitis is probably the most common acute CNS demyelinating disorder, preferentially affecting younger adults and children. Usually, symptoms include a viral prodrome, followed by an acute or subacute encephalopathy, visual defects, polyfocal motor and sensory deficits, and symptomatic seizures [5]. ADEM lesions can involve both the white (WM) and gray matter (GM), affecting the subcortical and central WM, cortical GM-WM junction and deep GM of the brainstem, cerebellum thalami, and basal ganglia [6]. A rising number of reports of COVID-19-associated ADEM in adults have been recently published. Langley et al. reported a 53-year-old male who was diagnosed with ADEM two months after severe COVID-19 infection, suggesting a possible delay in clinical presentation [7]. A case series by Paterson et al. described 24 patients with COVID-19-associated neurological disorders; out of them, nine patients were diagnosed with ADEM [8]. Trials of viral isolation from CSF samples are usually unsuccessful indicating a lack of direct viral invasion, although CSF proteins are typically elevated [9].

Literature is sparser with regard to pediatric COVID-19-associated ADEM. Assuncao et al. reported a nine-year-old who presented with difficulty walking, speech alteration, and right hemiparesis without concomitant respiratory symptoms [10]. His brain MRI showed multiple large hyperintense oval lesions in the subcortical white matter, internal capsule, and posterior fossa structures. He tested positive for COVID-19 PCR and was thus diagnosed with COVID-19-attributed ADEM [10]. Henriques et al. described a 12-year-old girl who had ADEM and extensive cervical spinal cord myelopathy with concomitant COVID-19 infection and near full recovery [11]. Younger patients were also reported, such as a 17-month-old who had developed ADEM two weeks after COVID-19 pneumonitis and was able to ultimately achieve full recovery after an intensive immunomodulatory treatment course [12]. Interestingly, acute systemic COVID-19 infection seems to exacerbate pre-existing ADEM symptoms in some patients [13]. Prognosis can vary, and out of 38 pediatric patients with neuro-COVID-19, four had rapid demise due to fulminant systemic co-infections, with one patient having a presumed diagnosis of ADEM [14].

Transverse myelitis (TM) is a focal inflammatory disorder affecting the spinal cord and presenting with rapid onset weakness, sensory deficits, and bowel and bladder dysfunction. COVID-19-attributed TM in children is thought to be very rare. In a review of 43 patients with COVID-19-associated acute TM, 40 were adults and 23 were male, with the cervicothoracic spinal cord being the most commonly affected segment [15]. Kaur et al. had probably reported the youngest published patient of a three-year-old female with COVID-19-associated TM [16]. More rarely, COVID-19-associated TM patients may have extraspinal symptoms such as dysautonomia [17] or concomitant Guillain Barre Syndrome [18]. Of note, and given the recent introduction of the COVID-19 vaccine for children, the possibility of post-vaccination TM is to be considered as well [19].

Neuromyelitis optica spectrum disorder (NMOSD) is a rare, debilitating neuroinflammatory disorder, which typically presents with recurrent optic neuritis (ON) and TM but also includes other closely related clinical syndromes such as acute brainstem syndrome (ABS), diencephalic syndrome, and area postrema syndrome (APS) [20]. The detection of AQP4-IgG often supports the diagnosis, and children are particularly rarely affected. Viral infections are thought to facilitate the replication and crossover of AQP4 IgG antibodies through the blood-brain barrier in most patients [20]. Batum et al reported a 50-year-old male with post-COVID-19 infection C3 myelitis, optic neuritis, and AQP4 positivity [21]. Ghosh et al. reported a 20-year-old male with APS, longitudinally extensive TM, and seropositivity for AQP4 antibody following acute COVID-19 pneumonia [22]. Ruijter et al. reported the first pediatric patient in his publication of a 15-year-old, post-COVID-19 male who presented with subacute vision loss and was found to be positive for AQP4 and SARS-CoV-2 PCR [23].

Myelin oligodendrocyte glycoprotein (MOG) antibody-associated disorders constitute an unfolding subgroup of CNS demyelinating disorders in children with monophasic or relapsing, yet overlapping, clinical features and the involvement of optic, cerebral, and spinal structures [24]. Optic neuritis is the most commonly reported symptom but the correlation between clinical severity and the presence or titers of MOG antibodies is questionable. The disorder appears to be more prevalent in children and young adults. However, a 63-year-old with para COVID-19 and seropositive MOG was recently reported [25]. Zhou et al. reported the first case of a 26-year-old who presented with dry cough and acute vision loss, to be ultimately diagnosed with COVID-19-associated, MOG-positive optic neuritis [26]. The first pediatric patient was recently published by Khat et al. of an 11-year-old male with bilateral optic neuritis and CSF MOG positivity [27]. Rarely, COVID-19 infection can also serve as a trigger for anti-MOG disease relapse [28].

Multiple sclerosis (MS) is the most prevalent entity of chronic CNS demyelinating disorders. About 10% of patients are children although only a handful of treatment options have been approved due to the rarity of clinical trials [29]. Immune system hyperreactivity seems to characterize COVID-19-associated multisystem inflammatory syndrome in children (MIS-C) and hence the emergence of autoantibodies in this particular population [30]. Although other coronavirus infections are thought to be known triggers, having the first MS presentation along with acute COVID-19 infection is exceptionally rare [31]. Fragoso & Palao et al. described a 27-year-old and 29-year-old woman with new-onset MS diagnosed a few weeks following acute COVID-19 infection [32-33]. At the time of writing this paper, the authors are not aware of any published reports of COVID-19-induced new-onset MS in children.

Although children, in general, appear to exhibit a milder COVID-19 infection phenotype, some special pediatric populations with preexisting medical issues may have a different risk profile [34]. Adults and children with chronic demyelinating disorders, and particularly those taking disease-modifying therapies, are at a higher risk of a severe COVID-19 phenotype and complications [35]. Younger age, the nature of the existing medical disorder, history of associated brain dysmaturity, and the severity of comorbidities may all act synergistically to increase such risks [36]. Moreover, children with prior neurological conditions, including demyelinating disorder, may be especially vulnerable because of delayed symptoms reporting, the lag time in seeking medical advice, difficulty with medical access, and ongoing challenges with health disparities [37]. There have been few guideline revisions and clinical care recommendations for disease-specific care for adults with a chronic demyelinating disorder during the pandemic, however, such guidelines are lacking when it comes to the pediatric age group [38]. Knowledge related to the safety, tolerability, and efficiency of the various forms of COVID-19 vaccines is still unfolding, although there is a consensus about vaccine benefits outweighing potential risks [39]. From a public health standpoint and considering the potential vector role of children in disease transmission, it will be interesting to see if data regarding children with MS and other demyelinating disorders will ultimately support evidence-based benefits [40].

## Conclusions

Childhood-onset CNS demyelinating disorders are rare, immune-mediated pathological phenomena, presumably triggered by minor viral infections in the pediatric age group. COVID-19 infection can be associated with the emergence of those disorders in children, but available data are sparse. We report a unique case series of five children with COVID-19 infection that is associated with various variants of demyelinating disorders. Although a causative relationship cannot be established yet, raising awareness of such a potential correlation can have a significant impact on the diagnosis and management of those patients. We believe that reports of this type will help improve the cumulative understanding of the neurological sequelae of the SARS-CoV-2 virus, which may last beyond the current worldwide pandemic.

## References

[1] Asadi-Pooya AA, Simani L (2020). Central nervous system manifestations of COVID-19: a systematic review. J Neurol Sci.

[2] Wu Y, Xu X, Chen Z (2020). Nervous system involvement after infection with COVID-19 and other coronaviruses. Brain Behav Immun.

[3] Torisu H, Okada K (2019). Vaccination-associated acute disseminated encephalomyelitis. Vaccine.

[4] Desforges M, Le Coupanec A, Dubeau P, Bourgouin A, Lajoie L, Dubé M, Talbot PJ (2019). Human coronaviruses and other respiratory viruses: underestimated opportunistic pathogens of the central nervous system?. Viruses.

[5] Filippi M, Rocca MA (2020). Acute disseminated encephalomyelitis. White Matter Diseases. An Update for Neurologists.

[6] Reichard RR, Kashani KB, Boire NA, Constantopoulos E, Guo Y, Lucchinetti CF (2020). Neuropathology of COVID-19: a spectrum of vascular and acute disseminated encephalomyelitis (ADEM)-like pathology. Acta Neuropathol.

[7] Langley L, Zeicu C, Whitton L, Pauls M (2020). Acute disseminated encephalomyelitis (ADEM) associated with COVID-19. BMJ Case Rep.

[8] Paterson RW, Brown RL, Benjamin L (2020). The emerging spectrum of COVID-19 neurology: clinical, radiological and laboratory findings. Brain.

[9] McCuddy M, Kelkar P, Zhao Y, Wicklund D (2020). Acute demyelinating encephalomyelitis (ADEM) in COVID-19 infection: a case series. Neurol India.

[10] Assunção FB, Fragoso DC, Donoso Scoppetta TL, Martins Maia AC (2021). COVID-19-associated acute disseminated encephalomyelitis-like disease. AJNR Am J Neuroradiol.

[11] de Miranda Henriques-Souza AM, de Melo AC, de Aguiar Coelho Silva Madeiro B, Freitas LF, Sampaio Rocha-Filho PA, Gonçalves FG (2021). Acute disseminated encephalomyelitis in a COVID-19 pediatric patient. Neuroradiology.

[12] McLendon LA, Rao CK, Da Hora CC, Islamovic F, Galan FN (2021). Post-COVID-19 acute disseminated encephalomyelitis in a 17-month-old. Pediatrics.

[13] Hussein O, Abd Elazim A, Torbey MT (2020). Covid-19 systemic infection exacerbates pre-existing acute disseminated encephalomyelitis (ADEM). J Neuroimmunol.

[14] Lindan CE, Mankad K, Ram D (2021). Neuroimaging manifestations in children with SARS-CoV-2 infection: a multinational, multicentre collaborative study. Lancet Child Adolesc Health.

[15] Román GC, Gracia F, Torres A, Palacios A, Gracia K, Harris D (2021). Acute transverse myelitis (ATM): clinical review of 43 patients with COVID-19-associated ATM and 3 post-vaccination ATM serious adverse events with the ChAdOx1 nCoV-19 vaccine (AZD1222). Front Immunol.

[16] Kaur H, Mason JA, Bajracharya M (2020). Transverse myelitis in a child with COVID-19. Pediatr Neurol.

[17] Moreno-Escobar MC, Kataria S, Khan E (2021). Acute transverse myelitis with dysautonomia following SARS-CoV-2 infection: a case report and review of literature. J Neuroimmunol.

[18] Khera D, Didel S, Panda S, Tiwari S, Singh K (2021). Concurrent longitudinally extensive transverse myelitis and Guillain-Barré syndrome in a child secondary to COVID-19 infection. A severe neuroimmunologic complication of COVID-19. Pediatr Infect Dis J.

[19] Artemiadis A, Liampas A, Hadjigeorgiou L, Zis P (2021). Myelopathy associated with SARS-COV-2 infection. A systematic review. Neurol Res.

[20] Huda S, Whittam D, Bhojak M, Chamberlain J, Noonan C, Jacob A (2019). Neuromyelitis optica spectrum disorders. Clin Med (Lond).

[21] Batum M, Kisabay Ak A, Mavioğlu H (2020). Covid-19 infection-induced neuromyelitis optica: a case report. Int J Neurosci.

[22] Ghosh R, De K, Roy D (2020). A case of area postrema variant of neuromyelitis optica spectrum disorder following SARS-CoV-2 infection. J Neuroimmunol.

[23] de Ruijter NS, Kramer G, Gons RA, Hengstman GJ (2020). Neuromyelitis optica spectrum disorder after presumed coronavirus (COVID-19) infection: a case report. Mult Scler Relat Disord.

[24] de Mol CL, Wong Y, van Pelt ED (2020). The clinical spectrum and incidence of anti-MOG-associated acquired demyelinating syndromes in children and adults. Mult Scler.

[25] Žorić L, Rajović-Mrkić I, Čolak E, Mirić D, Kisić B (2021). Optic neuritis in a patient with seropositive myelin oligodendrocyte glycoprotein antibody during the post-COVID-19 Period. Int Med Case Rep J.

[26] Zhou S, Jones-Lopez EC, Soneji DJ, Azevedo CJ, Patel VR (2020). Myelin oligodendrocyte glycoprotein antibody-associated optic neuritis and myelitis in COVID-19. J Neuroophthalmol.

[27] Khan A, Panwala H, Ramadoss D, Khubchandani R (2021). Myelin oligodendrocyte glycoprotein (MOG) antibody disease in a 11 year old with COVID-19 infection. Indian J Pediatr.

[28] Woodhall M, Mitchell JW, Gibbons E, Healy S, Waters P, Huda S (2020). Case report: myelin oligodendrocyte glycoprotein antibody-associated relapse with COVID-19. Front Neurol.

[29] Yeshokumar AK, Narula S, Banwell B (2017). Pediatric multiple sclerosis. Curr Opin Neurol.

[30] Lin JE, Asfour A, Sewell TB (2021). Neurological issues in children with COVID-19. Neurosci Lett.

[31] Moore L, Ghannam M, Manousakis G (2021). A first presentation of multiple sclerosis with concurrent COVID-19 infection. eNeurologicalSci.

[32] Fragoso YD, Pacheco FA, Silveira GL, Oliveira RA, Carvalho VM, Martimbianco AL (2021). COVID-19 in a temporal relation to the onset of multiple sclerosis. Mult Scler Relat Disord.

[33] Palao M, Fernández-Díaz E, Gracia-Gil J, Romero-Sánchez CM, Díaz-Maroto I, Segura T (2020). Multiple sclerosis following SARS-CoV-2 infection. Mult Scler Relat Disord.

[34] Bellino S, Punzo O, Rota MC (2020). COVID-19 disease severity risk factors for pediatric patients in Italy. Pediatrics.

[35] Sharifian-Dorche M, Sahraian MA, Fadda G (2021). COVID-19 and disease-modifying therapies in patients with demyelinating diseases of the central nervous system: a systematic review. Mult Scler Relat Disord.

[36] Graff K, Smith C, Silveira L (2021). Risk factors for severe COVID-19 in children. Pediatr Infect Dis J.

[37] Boronat S (2020). Neurologic care of COVID-19 in children. Front Neurol.

[38] Salter A, Fox RJ, Newsome SD (2021). Outcomes and risk factors associated with SARS-CoV-2 infection in a North American registry of patients with multiple sclerosis. JAMA Neurol.

[39] Kelly H, Sokola B, Abboud H (2021). Safety and efficacy of COVID-19 vaccines in multiple sclerosis patients. J Neuroimmunol.

[40] Zimet GD, Silverman RD, Fortenberry JD (2021). Coronavirus disease 2019 and vaccination of children and adolescents: prospects and challenges. J Pediatr.

